# Long non-coding RNA MALAT1 drives gastric cancer progression by regulating HMGB2 modulating the miR-1297

**DOI:** 10.1186/s12935-017-0408-8

**Published:** 2017-04-06

**Authors:** Jijun Li, Jinghua Gao, Wen Tian, Yongsheng Li, Jinghua Zhang

**Affiliations:** Department of Medical Oncology, Hebei Cang Zhou Central Hospital, No.16, Xinhuaxi Road, Cangzhou, Hebei 061000 China

**Keywords:** Gastric cancer (GC), Long non-coding RNA, MALAT1, miR-1297, HMGB2

## Abstract

**Background:**

Emerging evidences have verified that long non-coding RNAs (lncRNAs) play important regulatory roles in the pathogenesis and progression of cancers. lncRNAs metastasis associated lung adenocarcinoma transcript 1 (MALAT1) have been found to be up-regulated in some human cancers. The main objective of this study was to investigate the expression level and biological function of MALAT1 in gastric cancer (GC).

**Methods:**

Quantificational real-time polymerase chain reaction (qRT-PCR) was performed to detect the mRNA levels of MALAT1 in 78 paired gastric carcinoma tissues and adjacent normal tissues, and the associations of MALAT1 expression with the clinicopathological features were analyzed, and the prognosis of gastric carcinoma patients was evaluated. The HMGB2 mRNA and protein expressions were detected by qRT-PCR and western-blot analysis. Luciferase reporter assay was used to determine miR-1297 was a target of MALAT1.

**Results:**

In this study, we demonstrated MALAT1 was up-regulation in GC tissues compared with adjacent normal tissues and higher MALAT1 expression was correlated with local invasion, lymph node metastasis and TNM stage. Patients with higher MALAT1 expression predicted a shorter survival and poor prognosis. Functionally, we revealed that MALAT1 promoted cells proliferation and invasion in GC. Mechanistically, our results demonstrated that MALAT1 was negatively correlation with miR-1297 and functioned as a molecular sponging miR-1297, antagonizing its ability to suppress HMGB2 expression.

**Conclusions:**

Taken together, these results demonstrated that MALAT1/miR-1297/HMGB2 axis acted as critical regulator pathway in GC tumorigenesis and progression, which provided a novel therapeutic target for gastric cancer.

## Background

Gastric cancer is the most common form of gastrointestinal cancer and a leading cause of cancer-related mortality worldwide [1]. Despite a marked decrease incidence, especially in mortality rate in many countries, the absolute number of gastric cancer cases and deaths is still a big burden of the local health program [2]. Although some advancements in diagnostic and therapeutic including surgery, chemotherapy, and radiotherapy, due to the risk of relapse, distant metastasis, and chemoresistance, the 5-year survival rate in GC patients remains larger unsatisfied [3]. Thus, to explore more molecular biomarkers for predicting responsiveness of treatment, tumor progression, and potential target therapies are becoming more urgent.

Long-non-coding RNA (LncRNAs), which are >200 nt in length, play important roles in diverse biological processes, including cell proliferation, cell apoptosis, cell differentiation, cell invasion, and metastasis by regulating gene expression at the epigenetic, transcriptional, and posttranscriptional levels [4]. Alterations in long non-coding RNAs (lncRNAs) are associated with human carcinogenesis including gastric cancer. For instance, long non-coding RNA PVT1 indicated a poor prognosis of gastric cancer and promoted cell proliferation through epigenetically regulating p15 and p16 [5]. Plasma level of H19 was significantly higher in GC patients and could serve as a potential biomarker for diagnosis of GC, in particular for early tumor screening [6]. MEG3 was decreased in GC patients and MEG3 could up-regulated Bcl-2 via its competing endogenous RNA (ceRNA) activity on miR-181a [7]. Long non-coding RNA HOXA-AS2 promoted gastric cancer proliferation by epigenetically silencing P21/PLK3/DDIT3 expression [8]. These studies suggest an important role for MALAT1 in tumor biology.

Increasing evidences have shown that the aberrant expression of MALAT1 played a crucial role in carcinogenesis [9]. Such as, previous studies suggested that long non-coding RNA MALAT1 promoted tumor growth and metastasis by inducing epithelial-mesenchymal transition in oral squamous cell carcinoma [10]. MALAT1 increased AKAP-9 expression by promoting SRPK1-catalyzed SRSF1 phosphorylation in colorectal cancer cells [11]. Wang et al. reported that MALAT1 promoted malignant development of esophageal squamous cell carcinoma by targeting β-catenin via Ezh2 [12]. In GC, Metastasis-associated long non-coding RNA drove gastric cancer development and promoted peritoneal metastasis and as a novel therapeutic target in patients with gastric neoplasia [13]. Qi and his assistants also confirmed that MALAT1 bound EZH2, suppressed the tumor suppressor PCDH10, and promotes gastric cellular migration and invasion [14]. However, the biological role of MALAT1 in GC and the underlying molecular mechanism still remain undefined.

In the study, our results demonstrated MALAT1 was up-regulation in GC tissues and patients with higher MALAT1 expression had a shorter survival and poor prognosis. Further experiments demonstrated that knockdown of MALAT1 inhibited the GC cells proliferation and invasion. Mechanistically, we found that MALAT1 functioned as a molecular sponge for miR-1297, antagonizing its ability to inhibit HMGB2 protein expression. To sum up, our findings revealed that MALAT1 might function as a competing endogenous RNA (ceRNA) sponge for miR-1297 modulating HMGB2 expression. The MALAT1/miR-1297/HMGB2 regulator pathway provided a novel therapeutic method for gastric cancer.

## Methods

### Patients and tissue samples

Human GC tissues and adjacent non-cancerous tissues were collected from 78 GC patients who underwent surgical resection and obtained from Hebei Cangzhou Central Hospital. The diagnosis of all GC patients was histopathologically confirmed by two professional pathologists. None of the patients had received radiotherapy or chemotherapy before surgery. Written informed consent was obtained from all patients or their families for their related tissues were used. The approval for this study was obtained from Research Ethics Board at Hebei Cangzhou Central Hospital.

### Cell culture

The human GC cell lines MKN45, MKN28, BGC-823, and SGC-7901 and an immortalized normal gastric epithelial cell line GES-1 were purchased from the Cell Bank of the Chinese Academy of science. All cells were cultured in RPMI 1640 medium (GIBCO, Carlsbad, CA) with 10% FBS and 5% CO_2_ at 37 °C.

### QRT-PCR analysis

Total RNA from tissues and GC cells was extracted using Trizol reagent (Invitrogen, Carlsbad, USA). The One Step SYBR^®^ Prime Script^®^ PLUS RT-RNA PCR Kit (TaKaRa, Dalian, China) was used for the Real-Time PCR analysis. All experiments were repeated three times. GAPDH or U6 was used as the endogenous control. The relative fold changes in the transcripts were calculated using the 2^−ΔΔCt^ method. The QRT-PCR was performed at 95 °C for 15 s, followed by 40 cycles of 95 °C for 5 s and 60 °C for 34 s at ABI7500 real-time PCR detection system. The primer sequences used were as follows: for GAPDH-forward, 5′-GTCAACGGATTTGGTCTGTATT-3′andGAPDH-reverse, 5′-AGTCTTCTGGGTGGCAGTGAT-3′; MALAT1-forward5′-ATGCGAGTTGTTCTCCGTCT-3′and MALAT1-reverse, 5′-TATCTGCGGTTTCCTCAAGC-3′.

### Cell transfection assays

Cell transfections were performed using the Lipofectamine 3000 kit (Invitrogen) according to the manufacturer’s instructions. To knockdown MALAT1, the two siRNA oligonucleotides targeting MALAT1 and negative control were purchased from GenePharma (Shanghai, China) and were transfected into GC cells. The sequences of the targeting MALAT1were si-RNA1 sense-1,5′-CACAGGGAAAGCGAGTGGTTGGTAA-3′antisense-1,5′-TTACCAACCACTCGCTTTCCCTGTG-3′; si-RNA-2, sense-2, 5′-GAGGUGUAAAGGGAUUUAUTT-3′; antisense-2, 5′-AUAAAUCCCUUUACACCUCTT-3′). The working concentration of relative plasimids was 100 nM. MiR-1297 plasmid was transfected into GC cells using Lipofectamine TM 3000 (Invitrogen, Carlsbad, CA, USA).

### Cell proliferation and invasion assays

Cell proliferation was measured using the Cell Counting Kit-8 (CCK-8). Briefly, 3000 MKN45 or MKN28 cells/well were seeded into a 96-well plate. At daily intervals of 0, 24, 48, and 72 h, the optical density was measured at 450 nm using a microtiter plate reader (Quant BioTek Instruments). The cell invasion assays was performed according to the previously described using Transwell Chamber Cell Culture (8-μm pore membrane, BD Biosciences) [15]. The 1 × 10^5^ cells GC cells were added to the top chamber of 24-well plates with serum-free medium. The bottom chamber was added medium with 10% FBS. Cells in chamber were fixed with methanol for 20 min and then staining with Crystal violet for 10 min. Invaded cells were finally observed under a microscope and the number was counted. The results represent the average of three replicates.

### Western blotting assays

Cells were lysed in RIPA buffer containing fresh protease and phosphatase inhibitor cocktails (Sigma). Protein was separated by 10% SDS–PAGE and was transferred to PVDF membranes. The membranes were blocked in 5% skim milk and incubated with anti-HMGB2 antibody (1:1000 dilution, CST, USA), anti-PCNA antibody (1:3000 dilution, Abcam, CST, USA), anti-Ki67 antibody (1:1000 dilution, CST, USA) and anti-GAPDH antibody (1:2000 dilution, CST, USA) and incubated overnight at 4 °C. GAPDH served as an endogenous control and incubated overnight at 4 °C. Then, Horseradish peroxidase (HRP)-conjugated secondary antibody (1:1000 dilution, Abcam, USA) was incubated at room temperature for 2 h. Blots were developed using enhanced chemiluminescence detection reagents and scanned with a Molecular Imager system (Bio-Rad). Three repeated experiments were performed.

### Luciferase reporter assay

PMIRGLO-MALAT1 wild type (WT) or pMIRGLO-MALAT1 mutant type (MUT) reporter plasmid was co-transfected with miR-1297 mimic or NC mimic into MKN45 cells. Reporter plasmid and the internal control renilla luciferase plasmid were carried out with the appropriate plasmids using lipofectamine 3000 (Invitrogen, USA). The relative luciferase activity was normalized to renilla luciferase activity after transfection at 48 h, and luciferase activity was measured using a dual-luciferase reporter gene assay system (Promega, Madison, USA). Moreover, full-length MALAT1 by PCR was constructed and were transfected with a luciferase reporter vector containing perfect miR-1297 binding site (Sensor) wild type (WT) or mutant type (MUT) into theBCG-823 cells that expressed lower MALAT1 levels.

### Statistical analyses

All statistical analysis was performed using SPSS 16.0 software (version 16.0; Chicago, IL). Data are presented as mean ± standard deviation. Student’s *t* tests were used to evaluate significant differences between any two groups of data. One-way analysis of variance (ANOVA) was performed to analyze the differences between groups. Kaplan–Meier method and compared by log-rank test were used to construct survival curves. Pearson correlation analysis was used to detect the significance of association between MALAT1 and miR-1297 expression. The P < 0.05 was considered statistically significant.

## Results

### Expression of MALAT1 in gastric cancer tissues and cells

**Fig. 1:**
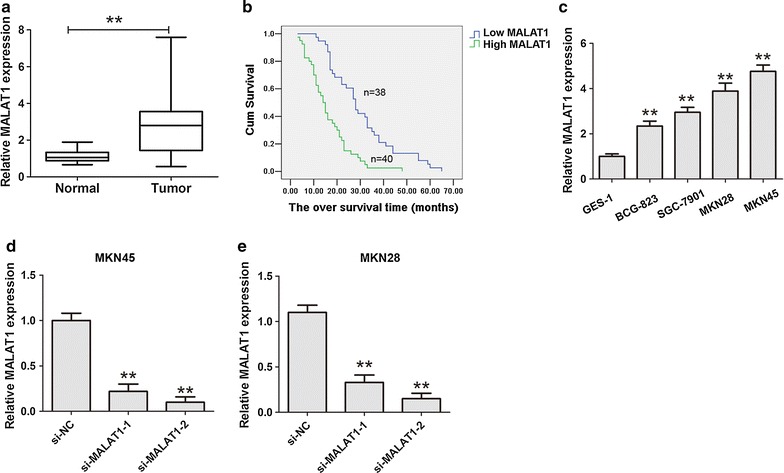
Up-regulation of MALAT1 in tumor tissues samples and cell lines. **a** The MALAT1 expression in 78 pairs of gastric cancer and corresponding non-cancerous gastric tissues was detected by qRT-PCR assays. **P < 0.05, the internal control was GAPDH. **b** Kaplan–Meier survival curve and log-rank test was perform to analyze the correlation between MALAT1 expression and the over survival (OS) time. **c** The MALAT1 expression in gastric cancer cells and an immortalized normal gastric epithelial cell line GES-1 was detected by qRT-PCR assays. **P < 0.05. **d**, **e** The MALAT1 expression in MKN45 or MKN28 cells was detected after knockdown of MALAT1 by qRT-PCR assays. Results were represented as the average ± SD based on 3 independent experiments. **P < 0.05

To investigate the MALAT1 expression in 78 paired GC tissues and adjacent non-cancer tissues, qRT-PCR analysis was applied. The results demonstrated that the expression of MALAT1 in GC tissues was up-regulated compared to the paired adjacent non-cancer tissues (Fig. 1a). We then analyzed the correlation between MALAT1 and Clinicopathological factors. As shown in Table 1, the result showed that MALAT1 expression was significantly correlation with local invasion, lymph node metastasis and TNM stage. Furthermore, we demonstrated that patients with higher MALAT1 expression had a shorter survival time in GC patients (Log-rank = 23.94, P < 0.001, Fig. 1b). As shown in Fig. 1c, we also demonstrated that MALAT1 expression was also significantly higher in four cell lines compared with that in GES-1 cells. The siRNA-2 was used to perform to silence the MALAT1 expression in the following experiences, according to knockout efficiency in the MKN45 and MKN28 cells (Fig. 1d, e).

**Table 1:** Association between MALAT1 expression levels and clinicopathologic parameters in 78 cases GC patients was evaluated

Clinicopathologic factors	MALAT1 expression levels			
	Patient number	MALAT1 lower	MALAT1 higher	P value
Gender				0.263
Male	42	24	18	
Female	36	16	20	
Age (years)				0.621
≤50	45	22	23	
>50	33	18	15	
Histological grade				0.879
High	19	9	10	
Middle	24	12	12	
Low	35	19	16	
Local invasion				0.003**
T1, T2	34	24	10	
T3, T4	44	16	28	
Lymph node metastasis				0.043**
No	43	27	18	
Yes	35	13	22	
Distant metastasis				0.086
No	50	22	28	
Yes	28	18	10	
TNM stage				0.039**
I/II	36	23	13	
III/IV	42	17	25	

** P < 0.05

### MiR-1297 is a direct target of MALAT1

**Fig. 2:**
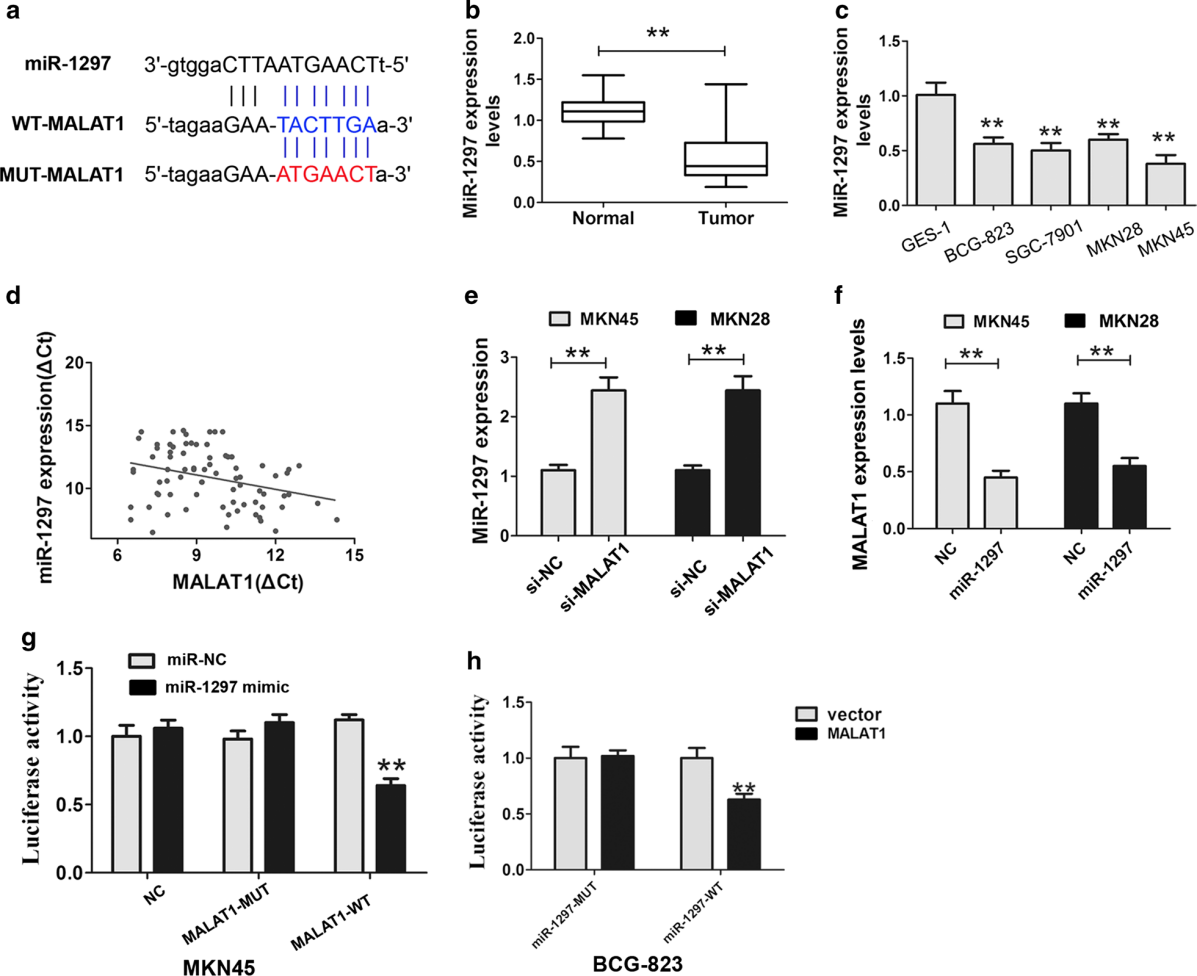
MALAT1 directly targeted miR-1297 in GC cells. **a** Bioinformatic analysis identified a potential miR-1297 target site of MALAT1 by software starbase (http://starbase.sysu.edu.cn/). **b** The miR-1297 expression levels in 78 pairs of gastric cancer and corresponding non-cancerous gastric tissues was detected by qRT-PCR assays. **P < 0.05, the internal control was U6, Student’s *t* test. **c** The miR-1297 expression in gastric cancer cells and an immortalized normal gastric epithelial cell line GES-1 was detected by qRT-PCR assays, the internal control was U6, **P < 0.05. **d** Pearson correlation analysis was used to detect the significance of association between MALAT1 and miR-1297 expression. **e** The miR-1297 expression in MKN45 and MKN28 cells was detected after MALAT1 silencing by qRT-PCR assays, the internal control was U6, **P < 0.05. **f** The MALAT1 expression in MKN45 or MKN28 cells was detected after transfecting miR-1297 by qRT-PCR assays, the internal control was GAPDH, **P < 0.05. **g** Co-transfection of miR-1297 mimic and PMIRGLO-MALAT1-WT strongly decreased the luciferase activity, while co-transfection of miR-1297 mimic and PMIRGLO-MALAT1-MUT and NC group did not change the luciferase activity, **h** Co-transfection of PmiRGLO-miR-1297-WT and MALAT1 overexpressed plasmid strongly decreased the luciferase activity, while co-transfection of PMIRGLO-miR-1297-MUT and MALAT1 overexpressed plasmid did not change the luciferase activity, Results were represented as the average ± SD based on 3 independent experiments. **P < 0.05

Recently, accumulating reports had indicated that MALAT1 functioned as ceRNAs to indirectly regulate miRNAs and hence functionally liberate other RNA transcripts [16]. To detect whether MALAT1 has a similar mechanism in GC cells, the online predicted bioinformatics software starbase (http://starbase.sysu.edu.cn/) was used to predict the potential mircroRNA binding sites in MALAT1. We found that miR-1297 had a binding site in MALAT1 (Fig. 2a). To further explore the potential correlation between MALAT1 and miR-1297, we examined the expression level of miR-1297 in GC tissues. Compared to the normal tissues, the expression of miR-1297 was markedly downregulated in GC tissues and cells (Fig. 2b, c). Furthermore,Pearson correlation analysis confirmed that MALAT1 expression level was negatively related to miR-1297 expression level in GC tissues (r = −0.317, P < 0.05, Fig. 2d). Next, we explored the expression of miR-1297 when MALAT1 was knocked down in MKN45 and MKN28 cells, and results demonstrated knockdown MALAT1 expression caused a significantly increase of miR-1297 expression (Fig. 2e). On the other hand, MALAT1 was significantly inhibited by transfecting the miR-1297 plasmid into MKN45 and MKN28 cells (Fig. 2f).

To confirm the direct binding relationship between MALAT1 and miR-1297, a luciferase activity assay was performed. The predicted miR-1297 binding site (MALAT1-WT) and its mutant type (MALAT1-MUT) were amplified and directly fused to the downstream of the luciferase reporter gene in the PMIRGLO-basic vector (Fig. 2a). The results showed that co-transfetion of miR-1297 and PMIRGLO-MALAT1-WT significantly decreased the luciferase activity, whereas co-transfection of miR-NC and PMIRGLO-MALAT1-MUT did not change the luciferace activity (Fig. 2g). Moreover, transfect with a luciferase reporter vector containing miR-1297 binding site-WT into the BCG-823 cells that expressed lower MALAT1 levels together with increasing amounts of a plasmid that expressed MALAT1 inhibited the miR-1297 binding site-WT luciferace activity (Fig. 2h). These results demonstrated that there exist a negative regulation between MALAT1 and miR-1297.

### MALAT1 regulates miR-1297 to modulate HMGB2 in gastric cancer cells

**Fig. 3:**
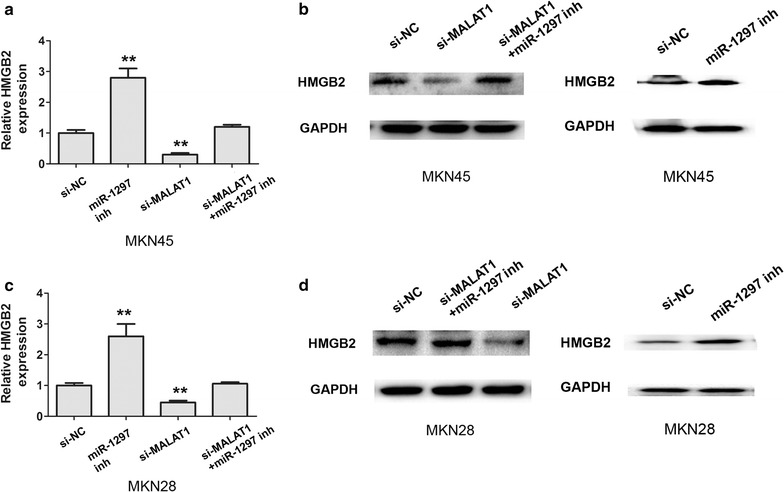
MALAT1 regulated miR-1297 to modulate HMGB2 in gastric cancer cells. **a**, **b** The mRNA and protein expression of HMGB2 after transfecting si-NC, si-MALAT1, si-MALAT1 and miR-1297 inhibitor in MKN45 cells was detected by qRT-PCR assays and western-blotting analysis, **P < 0.05. **c**, **d** The mRNA and protein expression of HMGB2 after transfecting si-NC, si-MALAT1, si-MALAT1 and miR-1297 inhibitor in MKN28 cells was detected by qRT-PCR assays and western-blotting analysis. Results were represented as the average ± SD based on 3 independent experiments. **P < 0.05

A previous study found that miR-1297 promoted apoptosis and inhibited the proliferation and invasion of hepatocellular carcinoma cells by targeting HMGA2 [17]. Thus, based on above result, we also investigated whether MALAT1 could regulate the expression of the miR-1297 targeting HMGB2. The results demonstrated that the mRNA and protein levels of HMGB2 were significantly decreased after knockdown of MALAT1 and was upregulated by miR-1297 inhibitor in the MKN45 cells, and the reduced expression of HMGB2 when knockdown of MALAT1 could be restored by co-transfecting with si-MALAT1 and miR-1297 inhibitor (Fig. 3a, b). Moreover, we also found that the mRNA and protein levels of HMGB2 were remarkably decreased after knockdown of MALAT1 in the MKN 28 cells,and this reduction could be restored by co-transfecting with si-MALAT1 and miR-1297 inhibitor (Fig. 3c, d). Our results confirmed that MALAT1 promoted HMGB2 through negatively regulating miR-1297.

### MALAT1 regulates miR-1297 to promote gastric cancer cell proliferation and invasion

**Fig. 4:**
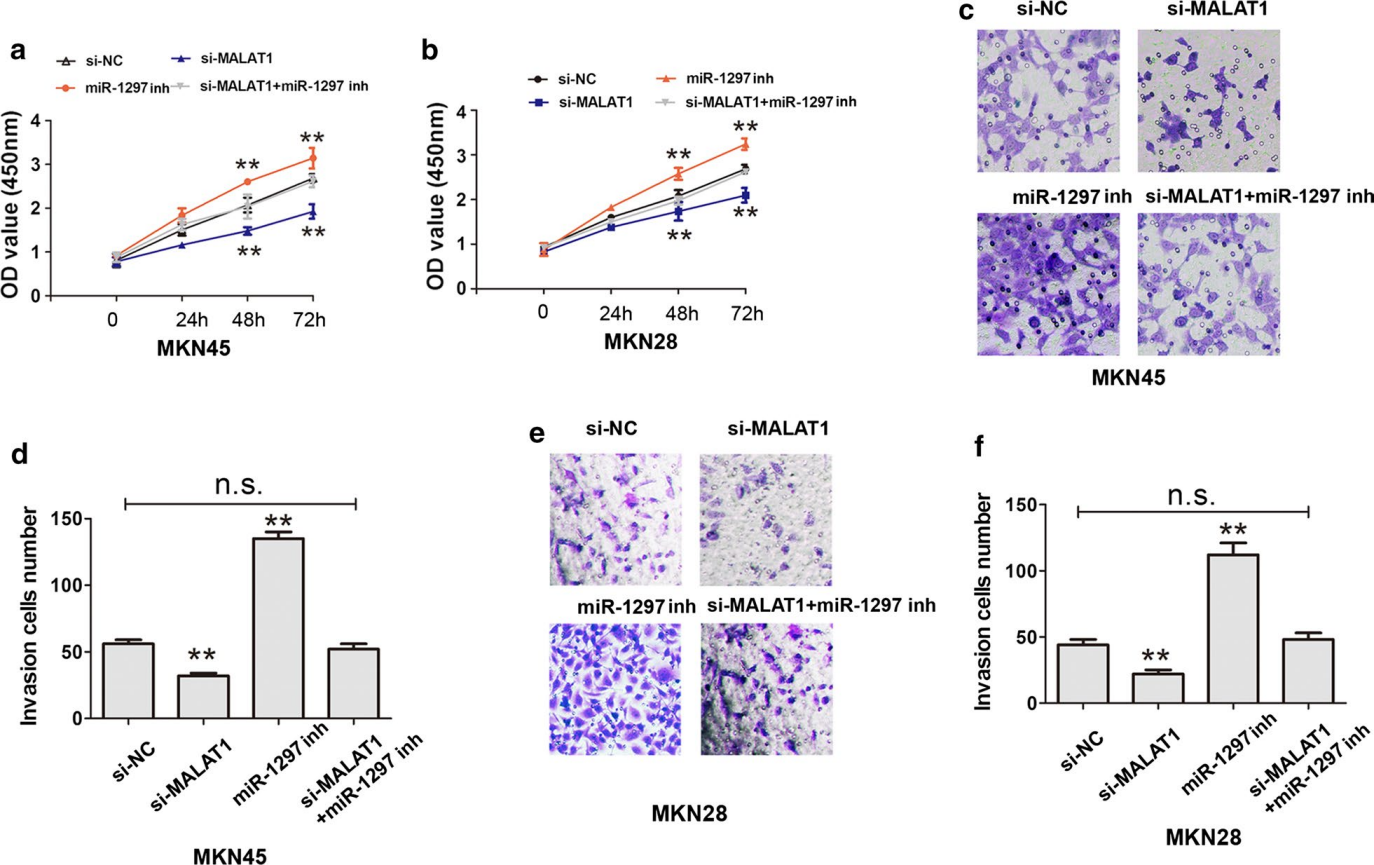
MALAT1 regulated miR-1297 expression and promoted gastric cancer cell proliferation and invasion. **a**, **b** CCK8 cell proliferation was performed to detect the cell abilities after MALAT1 down-regulation, and was rescued by miR-1297 inhibitor treatment in MKN45 or MKN28 cells, **P < 0.05. *n.s*. not statistics significant. **c**–**f** Transwell cell invasion assays and analysis were performed to detect the cell abilities after MALAT1 down-regulation, and were rescued by miR-1297 inhibitor treatment in MKN45 or MKN28 cells, **P < 0.05. *n.s*. not statistics significant. Results were represented as the average ± SD based on 3 independent experiments

**Fig. 5:**
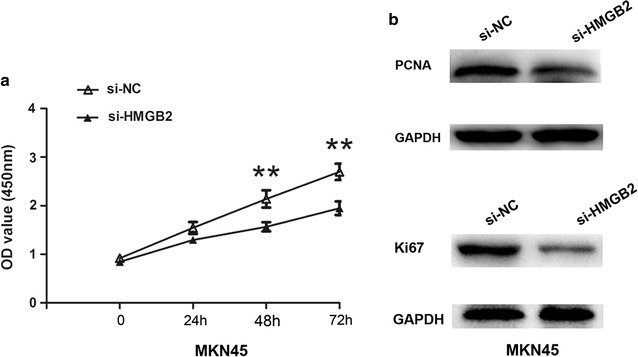
Knockdown of HGMB2 inhibited the GC cell proliferation. **a** CCK8 cell proliferation was performed to detect the cell abilities after HMGB2 down-regulation in MKN45 cells. **b** The protein expression of PCNA and Ki-57 was determined by western-blotting analysis in MKN45 cells. **P < 0.05, the results were represented as the average ± SD based on 3 independent experiments

Next, we examined MALAT1 effects on GC cell growth. CCK8 assays revealed that knockdown of significantly reduced the MKN45 and MKN28 cell proliferation abilities, and this reduction could be restored by co-transfecting with si-MALAT1 and miR-1297 inhibitor (Fig. 4a, b). In addition, transwell cell invasion assay also revealed that knockdown of MALAT1 efficiency inhibited the cell invasion abilities in MKN45 or MKN28 cells, and this inhibition could be restored by co-transfecting with si-MALAT1 and miR-1297 inhibitor (Fig. 4c, f). We further identified the underlying effects of HMGB2 on GC cell proliferation. CCK8 cell proliferation was performed to detect the cell growth abilities after HMGB2 silencing. The results showed that cell proliferation abilities were inhibited after HMGB2 silencing in MKN45 cell (Fig. 5a). Moreover, the expression of Proliferating Cell Nuclear Antigens (PCNA) and Ki-67 were inhibited after HMGB2 silencing in MKN45 cell (Fig. 5b, c). Therefore, the results demonstrated that MALAT1 negatively regulated miR-1297 and promote the HMGB2 expression in gastric cancer progression.

## Discussion

Recent research has revealed that MALAT1 play pivotal roles in tumorigenesis of cancers including gastric cancer [16, 18, 19]. MALAT1 was aberrantly highly expressed in GC cell lines and promoted cell proliferation in gastric cancer by recruiting SF2/ASF [20]. Plasma MALAT1 levels were significantly higher in gastric cancer patients with distant metastasis than patients without distant metastasis and the healthy controls [21]. In the study, we found the expression level of MALAT1 was significantly higher in gastric cancer tissues compared with adjacent normal tissues. Higher expression of MALAT1 was positively associated with local invasion, lymph node invasion and TNM stage. In addition, overexpression of MALAT1 was associated with short survival time. These results implicated that MALAT1 may play an important role in gastric cancer progression.

Previous reports have revealed that competing endogenous RNA (ceRNA) regulator mechanisms involved in MALAT1. For example, knockdown of long non-coding RNA MALAT1 increases the blood-tumor barrier permeability by up-regulating miR-140 [22]. MALAT1 induced migration and invasion of human breast cancer cells by competitively binding miR-1 with cdc42 [23]. Wang et al. reported that long non-coding RNA MALAT1 promoted gallbladder cancer cell proliferation and invasion targeting ANXA2 and KRAS by acting as a molecular sponge to regulate miR-206 [24]. Another study in lung adenocarcinoma demonstrated that lncRNA MALAT1 exerted oncogenic functions by targeting miR-204 [25]. It also been verified MALAT1 functioned as a competing endogenous RNA to regulate ZEB2 expression by sponging miR-200s in clear cell kidney carcinoma and promoted cell invasion and metastasis [26]. According to luciferase reporter assays results, co-transfection of miR-1297 and PMIRGLO- MALAT1-WT significantly decreased the luciferase activity. In vitro studies further confirmed that MALAT1 negatively regulated miR-1297 expression levels, which promoted cell proliferation and cell invasion. Moreover, miR-1297 was up-regulated after MALAT1 silencing, notably, ectopic expression of miR-1297 increased the MALAT1 expression levels. These findings demonstrated that there exist a negative regulation between MALAT1 and miR-1297.

Overexpression of high-mobility group box 2 was associated with tumor aggressiveness and prognosis of hepatocellular carcinoma [27]. Knockdown of HMGB2 decreased the chemoresistance of gastric cancer cells [28]. Our results showed that cell proliferation abilities were inhibited and PCNA and Ki-67 expression were reduced after HMGB2 silencing in MKN45 cell. Previous study showed that miR-1297 inducing HCC cell apoptosis and inhibited cell the proliferation and invasion by targeting HMGB2 [17]. In our study, knockdown of MALAT1 was able to reduce the mRNA and protein levels of HMGA2 and miR-1297 inhibitor could enhanced the HMGB2 expression. What’s more, knockdown of MALAT1 could reduce the mRNA and protein levels of HMGA2 which was dismissed by miR-1297 inhibitor. These data revealed that MALAT1 exerts its biological effects at least in part by modulating HMGA2 expression.

## Conclusions

In conclusion, we demonstrated that MALAT1 was significantly overexpressed in gastric cancer tissues and cell lines and revealed a MALAT1/miR-1297/HMGB2 regulator pathway in GC. Thus, our results indicated that intervention of MALAT1/miR-1297/HMGB2 regulator pathway could inhibit gastric cancer progression.

## Abbreviations

MALAT1: metastasis associated lung adenocarcinoma transcript 1
GC: gastric cancer
qRT-PCR: quantificational real-time quantitative polymerase chain reaction
GAPDH: glyceraldehyde-3-phosphate dehydrogenase
SDS-PAGE: sodium dodecyl sulfate-polyacrylamide gel electrophoresis
PVDF: polyvinylidene fluoride

## References

[1] Jemal A, Bray F, Center MM, Ferlay J, Ward E, Forman D (2011). Global cancer statistics. CA Cancer J Clin.

[2] Roder DM (2002). The epidemiology of gastric cancer. Gastric Cancer.

[3] Saka M, Morita S, Fukagawa T, Katai H (2011). Present and future status of gastric cancer surgery. Jpn J Clin Oncol.

[4] Wang J, Sun J, Wang J, Song Y, Gao P, Shi J, Chen P, Wang Z (2016). Long noncoding RNAs in gastric cancer: functions and clinical applications. Onco Targets Ther..

[5] Kong R, Zhang EB, Yin DD, You LH, Xu TP, Chen WM, Xia R, Wan L, Sun M, Wang ZX, De W, Zhang ZH (2015). Long noncoding RNA PVT1 indicates a poor prognosis of gastric cancer and promotes cell proliferation through epigenetically regulating p15 and p16. Mol Cancer..

[6] Zhou X, Yin C, Dang Y, Ye F, Zhang G (2015). Identification of the long non-coding RNA H19 in plasma as a novel biomarker for diagnosis of gastric cancer. Sci Rep..

[7] Peng W, Si S, Zhang Q, Li C, Zhao F, Wang F, Yu J, Ma R (2015). Long non-coding RNA MEG3 functions as a competing endogenous RNA to regulate gastric cancer progression. J Exp Clin Cancer Res..

[8] Xie M, Sun M, Zhu YN, Xia R, Liu YW, Ding J, Ma HW, He XZ, Zhang ZH, Liu ZJ, Liu XH, De W (2015). Long noncoding RNA HOXA-AS2 promotes gastric cancer proliferation by epigenetically silencing P21/PLK3/DDIT3 expression. Oncotarget..

[9] Tian X, Xu G (2015). Clinical value of lncRNA MALAT1 as a prognostic marker in human cancer: systematic review and meta-analysis. BMJ Open..

[10] Zhou X, Liu S, Cai G, Kong L, Zhang T, Ren Y, Wu Y, Mei M, Zhang L, Wang X (2015). Long non coding RNA MALAT1 promotes tumor growth and metastasis by inducing epithelial-mesenchymal transition in oral squamous cell carcinoma. Sci Rep..

[11] Hu ZY, Wang XY, Guo WB, Xie LY, Huang YQ, Liu YP, Xiao LW, Li SN, Zhu HF, Li ZG, Kan H (2016). Long non-coding RNA MALAT1 increases AKAP-9 expression by promoting SRPK1-catalyzed SRSF1 phosphorylation in colorectal cancer cells. Oncotarget..

[12] Wang W, Zhu Y, Li S, Chen X, Jiang G, Shen Z, Qiao Y, Wang L, Zheng P, Zhang Y (2016). Long noncoding RNA MALAT1 promotes malignant development of esophageal squamous cell carcinoma by targeting beta-catenin via Ezh2. Oncotarget..

[13] Okugawa Y, Toiyama Y, Hur K, Toden S, Saigusa S, Tanaka K, Inoue Y, Mohri Y, Kusunoki M, Boland CR, Goel A (2014). Metastasis-associated long non-coding RNA drives gastric cancer development and promotes peritoneal metastasis. Carcinogenesis.

[14] Qi Y, Ooi HS, Wu J, Chen J, Zhang X, Tan S, Yu Q, Li YY, Kang Y, Li H, Xiong Z, Zhu T, Liu B, Shao Z, Zhao X (2016). MALAT1 long ncRNA promotes gastric cancer metastasis by suppressing PCDH10. Oncotarget..

[15] Grieco P, Franco R, Bozzuto G, Toccacieli L, Sgambato A, Marra M, Zappavigna S, Migaldi M, Rossi G, Striano S, Marra L, Gallo L, Cittadini A, Botti G, Novellino E, Molinari A, Budillon A, Caraglia M (2011). Urotensin II receptor predicts the clinical outcome of prostate cancer patients and is involved in the regulation of motility of prostate adenocarcinoma cells. J Cell Biochem.

[16] Li T, Mo X, Fu L, Xiao B, Guo J (2016). Molecular mechanisms of long noncoding RNAs on gastric cancer. Oncotarget..

[17] Liu Y, Liang H, Jiang X (2015). MiR-1297 promotes apoptosis and inhibits the proliferation and invasion of hepatocellular carcinoma cells by targeting HMGA2. Int J Mol Med.

[18] Deng QJ, Xie LQ, Li H (2016). Overexpressed MALAT1 promotes invasion and metastasis of gastric cancer cells via increasing EGFL7 expression. Life Sci.

[19] Misso G, Zarone MR, Grimaldi A, Di Martino MT, Lombardi A, Kawasaki H, Stiuso P, Tassone P, Tagliaferri P, Caraglia M (2017). Non coding RNAs: a new avenue for the self-tailoring of blood cancer treatment. Curr Drug Targets.

[20] Wang J, Su L, Chen X, Li P, Cai Q, Yu B, Liu B, Wu W, Zhu Z (2014). MALAT1 promotes cell proliferation in gastric cancer by recruiting SF2/ASF. Biomed Pharmacother.

[21] Xia H, Chen Q, Chen Y, Ge X, Leng W, Tang Q, Ren M, Chen L, Yuan D, Zhang Y, Liu M, Gong Q, Bi F (2016). The lncRNA MALAT1 is a novel biomarker for gastric cancer metastasis. Oncotarget..

[22] Ma J, Wang P, Yao Y, Liu Y, Li Z, Liu X, Li Z, Zhao X, Xi Z, Teng H, Liu J, Xue Y (2016). Knockdown of long non-coding RNA MALAT1 increases the blood-tumor barrier permeability by up-regulating miR-140. Biochim Biophys Acta.

[23] Chou J, Wang B, Zheng T, Li X, Zheng L, Hu J, Zhang Y, Xing Y, Xi T (2016). MALAT1 induced migration and invasion of human breast cancer cells by competitively binding miR-1 with cdc42. Biochem Biophys Res Commun..

[24] Wang SH, Zhang WJ, Wu XC, Zhang MD, Weng MZ, Zhou D, Wang JD, Quan ZW (2016). Long non-coding RNA Malat1 promotes gallbladder cancer development by acting as a molecular sponge to regulate miR-206. Oncotarget..

[25] Li J, Wang J, Chen Y, Li S, Jin M, Wang H, Chen Z, Yu W (2016). LncRNA MALAT1 exerts oncogenic functions in lung adenocarcinoma by targeting miR-204. Am J Cancer Res..

[26] Xiao H, Tang K, Liu P, Chen K, Hu J, Zeng J, Xiao W, Yu G, Yao W, Zhou H, Li H, Pan Y, Li A, Ye Z, Wang J, Xu H, Huang Q (2015). LncRNA MALAT1 functions as a competing endogenous RNA to regulate ZEB2 expression by sponging miR-200 s in clear cell kidney carcinoma. Oncotarget..

[27] Kwon JH, Kim J, Park JY, Hong SM, Park CW, Hong SJ, Park SY, Choi YJ, Do IG, Joh JW, Kim DS (2010). Overexpression of high-mobility group box 2 is associated with tumor aggressiveness and prognosis of hepatocellular carcinoma. Clin Cancer Res.

[28] An Y, Zhang Z, Shang Y, Jiang X, Dong J, Yu P, Nie Y, Zhao Q (2015). miR-23b-3p regulates the chemoresistance of gastric cancer cells by targeting ATG12 and HMGB2. Cell Death Dis.

